# A Contribution to the History of the Respiration of Man

**Published:** 1898-03

**Authors:** 


**A Contribution to the History of the Respiration of Man.** By William Marcet, M.D., F.R.S. Pp. 116. London: J. & A. Churchill. 1897.

In a handsome volume, copiously illustrated with diagrams and charts, Dr. Marcet has reprinted, with additions, the four Croonian lectures which he delivered in 1895 before the Royal College of Physicians. They contain summaries of our present knowledge of the influence of oxygen on vital processes, of the action of diminished and increased atmospheric pressure on breathing, and of the effects of various volitional processes on respiration.

The book is one which will appeal to physiologists rather than to practitioners, for its practical bearing on clinical medicine is not very considerable.

